# Hepatitis A Immunity in the District of Aveiro (Portugal): An Eleven-Year Surveillance Study (2002–2012)

**DOI:** 10.3390/v6031336

**Published:** 2014-03-14

**Authors:** Sara Pereira, Inês Linhares, António Ferreira Neves, Adelaide Almeida

**Affiliations:** 1 Department of Biology and CESAM, University of Aveiro, Aveiro 3810-193, Portugal; E-Mails: sarasouto@ua.pt (S.P.); ineslinhares@ua.pt (I.L.); 2 Clinical Analysis Laboratory Avelab, Rua Cerâmica do Vouga, Aveiro 3800-011, Portugal; E-Mail: a.f.neves@netcabo.pt

**Keywords:** Hepatitis A, immunity, surveillance study, epidemiology, Portugal

## Abstract

Hepatitis A is a common viral liver disease and brings serious health and economic problems as its epidemiologic pattern changes over time. National serosurveys from developed countries have indicated a decline in HAV (hepatitis A virus) seroprevalence over time due to the improvement of economic and sanitation levels. The hepatitis A virus (HAV) immunity rate was surveyed throughout an eleven-year period by sex and age group in Aveiro District. In this retrospective study, blood samples from patients of Aveiro District, in ambulatory regime, collected at the Clinical Analysis Laboratory Avelab between 2002 and 2012 were screened for the presence of antibodies against HAV antigen using a chemiluminescence immunoassay. The global immunity (positive total anti-HAV) was 60% and only 0.3% of the patients presented recent infection by HAV (positive IgM anti-HAV). The HAV immunity was age-dependent (*p <* 0.05), but no significant differences (*p >* 0.05) between sexes were observed. The immunity was similar throughout the study period (*p >* 0.05). The results of this study indicate that young people (especially under 25 years old) from District of Aveiro are susceptible to HAV infection, constituting a high risk group. The elderly should be also a concern in the future of Hepatitis A infection.

## 1. Introduction

Hepatitis A is an acute disease transmitted by fecal-oral route, either by direct contact with an HAV-infected person or by ingestion of HAV-contaminated food or water. During the course of the disease there are three phases: incubation, symptomatic infection and convalescence and the HAV excretion occurs from the incubation to early symptomatic phase. The incubation period ranges from 15 to 50 days [1]. The main serological marker, IgM anti-HAV can be detected between five days and 6 months after exposure. Anti-HAV IgG confers lifelong immunity and this antibody can also be detectable in the symptomatic phase [2,3].

HAV causes liver disease worldwide, being annually estimated 1.4 million cases of new infections [4]. Despite the low mortality rate (between 0.1% and 2.1%), it causes a very significant morbidity [5]. HAV may also lead to extrahepatic complications (e.g., pancreatitis, vasculitis and glomerulonephritis). The main cause of death is fulminant hepatitis associated to chronic liver disease [6]. Hepatitis A causes epidemic waves that are usually repeated at intervals varying in accordance with the virus circulation [7].

In developed countries, the HAV incidence has declined essentially due to the great improvements of sanitary conditions [7]. However, a decrease of immune individuals leads to an increased risk of potential outbreaks. In such circumstances, these outbreaks may be unpredictable and difficult to control. This concern is also focused on the fact that, in children, hepatitis A is mostly asymptomatic. In adults, 70%–89% of cases are symptomatic and with increasing age the symptoms are worsened. Thus, although the incidence in population has decreased, there is an increase in costs per case of HAV infection as a large segment of the population is now susceptible to it [8]. There is an evidence that many of the outbreaks are misdiagnosed [9]. According to the World Health Organization (WHO), annual reports show about 100,000 cases of outbreaks in Europe, 500 of them being fatal. Knowledge of anti-HAV seroprevalence rates in the world and in each region of a country is of utmost importance to establish public health priorities and to adopt adequate vaccination policies [10].

In 2011, the WHO has compiled the immunity studies made across the world and a summary was made. In western countries, the overall immunity was 50%, and less than 20% to people aged less than 20 years. In this region, in which Portugal is included, there is a low child immunity rate and the adult susceptibility rate is high. In Central and Eastern Europe, there is a low-medium child immunity rate and the adult susceptibility rate is medium. In North America, there is a low child immunity rate and medium susceptibility in adults [10]. Between 1996 and 2006, 1164 patients were hospitalized with HAV in Portugal, 30% were younger than 15 years and 3% had severe liver failure (data of the Computing and Financial Management of Health) [11]. According to the largest epidemiological survey carried out in Portugal in 1984, comprising 1770 individuals distributed among several districts, the immunity rate for the Portuguese population was 84.9% and 93.4% to people aged less than 20 years. This country was considered highly endemic [12].

The vaccine is highly immunogenic, conferring protection against HAV in approximately 95% of the vaccinated patients [7]. Protection is considered to be lifelong after a complete hepatitis A vaccination schedule (two doses) [13,14]. A combined hepatitis A/B vaccine with high immunogenicity is also available in some countries [15]. The vaccine is not yet widely used but in more developed countries it is recommended for risk groups. Moreover, some studies have found that universal vaccination is cost-effective [7,16,17,18]. In Catalonia, the universal vaccination program of preadolescents began in 1998 and has avoided 90% of cases in young people aged 12–19 years [19]. In Portugal, the vaccine has been available since 1998 but is not yet widely used, and it is only recommended for specific at-risk groups. According to the national health regulatory authority (DGS) recommendations, children, adolescents or adults who travel to high or intermediate endemic countries, adolescents and adults with chronic liver disease or who belong to a community where an outbreak is detected should be primarily vaccinated [20]. The vaccine is not yet included in the National Plan of Vaccination [21].

The public health importance of this form of hepatitis is becoming increasingly recognized as the epidemiological picture of the disease has been dramatically changing in recent years. In many countries, outdated data are still being used in policy decisions [9]. Seroprevalence studies of hepatitis A as well as the evaluation of the cost-benefit of the vaccine may contribute to the decision of extending vaccination to the entire population. This study aims to evaluate the prevalence of hepatitis A in Aveiro District, from 2002 to 2012, in order to broaden knowledge on the epidemiology of this disease in Portugal and to assess the risk of outbreaks of HAV.

## 2. Results

### 2.1. Characterization of the Sample

**Figure 1:**
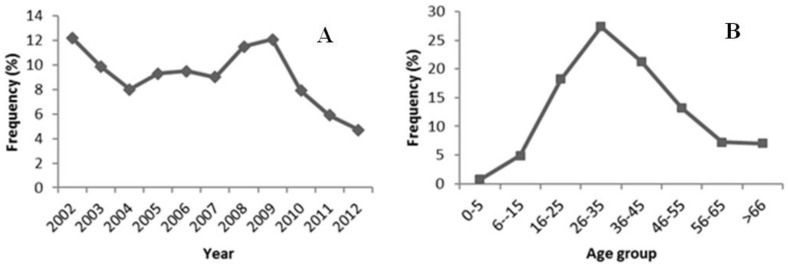
Frequency (%) of the samples by year (**A**) and age group (**B**) during the study period.

The annual average of analyzed samples was 718. Observations showed a decrease of about 7% in the number of analyses performed between 2009 and 2012 (Figure 1A). From the total of 7894 serum samples analyzed between 2002 and 2012, 4357 (55.2%) were performed in male patients and 3537 (44.8%) in females. The age of the patients ranged between 0 and 99 years. The patients aged between 26–35 years represented the age group that carried out more analysis. In general, an increase of the percentage of analysis performed until the age of 26–35 years was observed, meanwhile a decrease was found for patients aged more than 35 years (Figure 1B).

**Figure 2:**
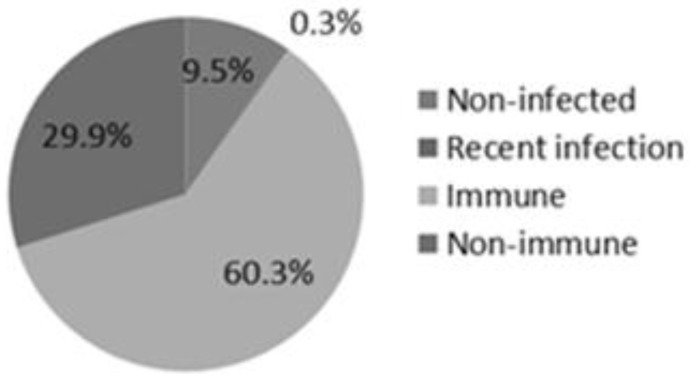
Sample classification during the study period.

From the 7894 serum samples analyzed, more than half of the patients (60.4%) had HAV immunity and only 0.3% of patients were recently infected with HAV (Figure 2). About 30% of the patients did not show immunity against HAV.

### 2.2. Characterization of HAV Immune Patients

**Figure 3:**
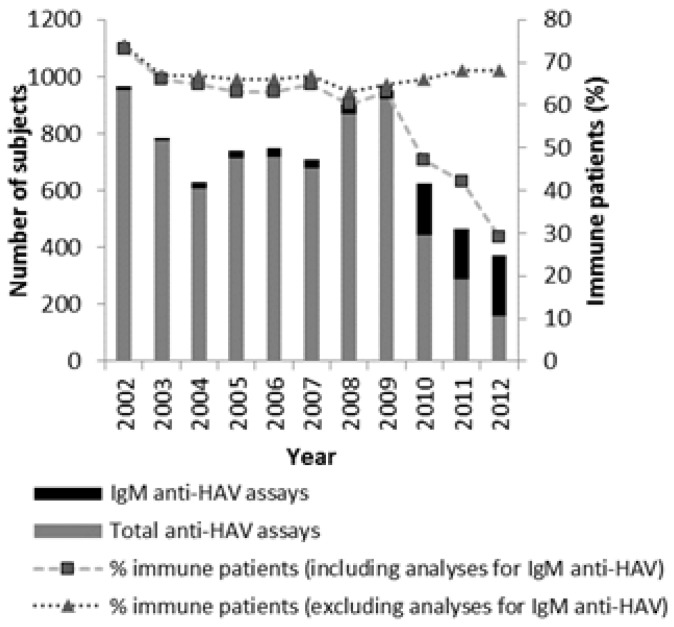
Samples classification during the study period**.**

There was an abrupt decrease in immunity from 2009 to 2012 but an inversion in the ratio of analysis required was also observed. Taking into account both analyses (IgM anti-HAV and total anti-HAV), the percentage of immunity was 73% (*n =* 966) in 2002, 63% (*n =* 953) in 2009 and 28.6% (*n =* 374) in 2012. However, for total anti-HAV detection, the percentage of immunity in 2002 was 73.6% (*n =* 954), in 2009 65.2% (*n =* 922) and in 2012, 67.7% (*n =* 158). Throughout the study period, slight changes in the incidence of the overall immunity were observed, but considering only the results of total anti-HAV the percentage of immunity did not vary significantly (*p >* 0.05) (Figure 3).

**Figure 4:**
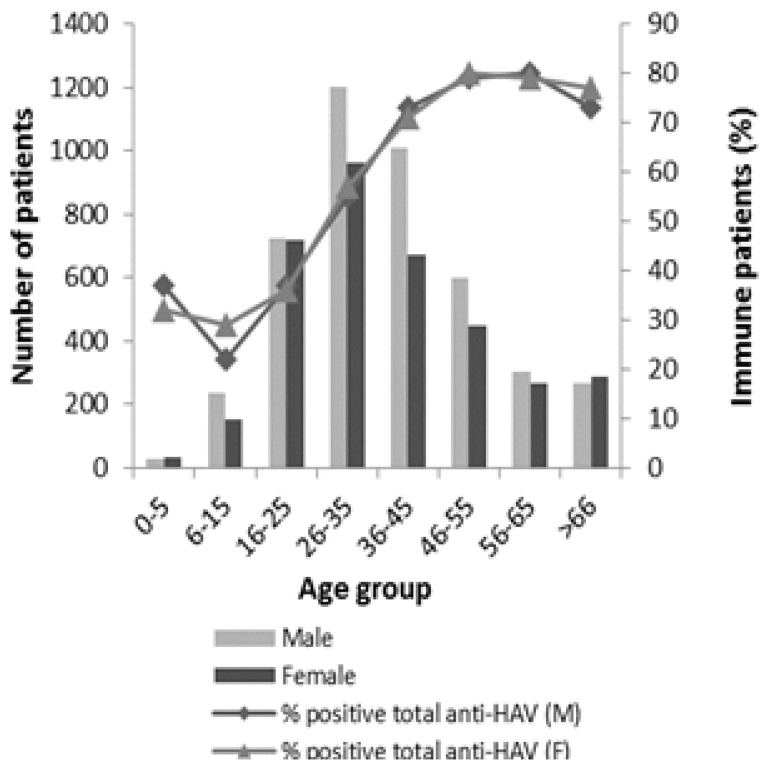
Percentage of immune patients by genre and age group. M–Male; F–Female.

The overall immunity was not significantly different between female and male patients (*p >* 0.05). However, significant differences in seroprevalence among the different age groups were observed (*p <* 0.05). The age of the immune patients ranged from 0 to 99 years old with a mean age of 42 years old. With the exception of patients aged less than five years, there was an overall increase of immunity with age. A gradually increase (54.8%) of immunity in patients with age comprised between 6–15 years and between 56–65 years was observed, in spite of a decrease of immunity of about 5% in patients aged between 56–65 and with more than 66 years. For patients aged over 25 years, the percentage of immunity was higher than 50% (Figure 4).

### 2.3. Characterization of Infected Patients

**Figure 5:**
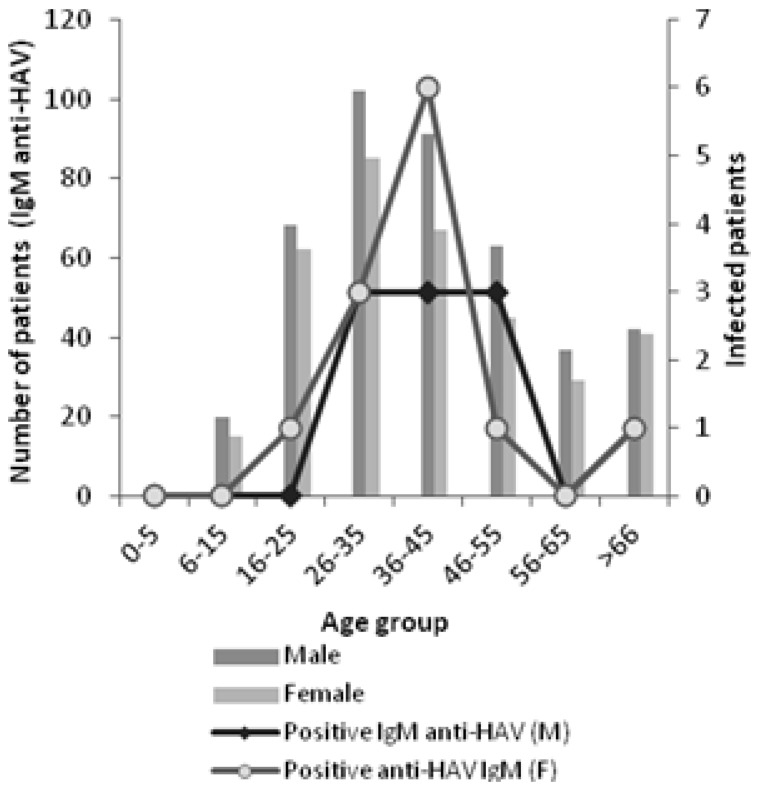
Number of infected patients by gender and age group.

**Figure 6:**
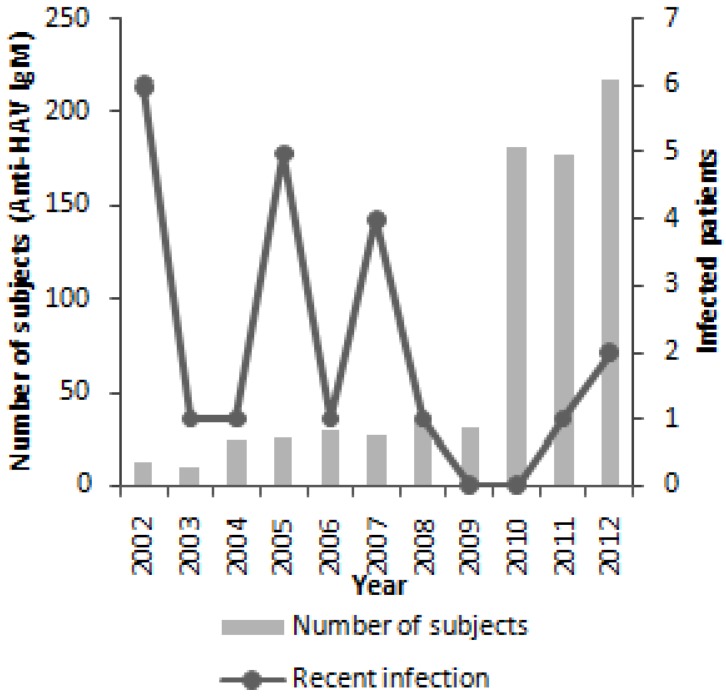
Number of infected patients by year.

The age of infected patients with positive IgM anti-HAV ranged from 22 to 87 years, with a mean age of 44 years. The IgM anti-HAV was detected in 22 patients (8 males and 12 females). The higher incidence of HAV infection was observed in patients aged from 36 to 45 years. The incidence of infection was not significantly different among age groups neither between genres (*p >* 0.05) (Figure 5), but varied over the study period (Figure 6).

## 3. Discussion

In this study, hepatitis A was examined in 7894 individuals throughout Aveiro District in order to evaluate the status of this viral infection, enriching the worldwide hepatitis A surveillance.

Having into account both currently used analyses (IgM anti-HAV and total anti-HAV), the percentage of immunity decreased during the study period but this decrease was result of a change in the type of analysis required by the patients. Before 2010 more than 90% of the samples were analyzed for total anti-HAV and after that there was a big decrease in its number (in 2012, total anti-HAV was analyzed in only 42% of the samples). This change led to doubtful results, since IgM anti-HAV does not allow us to survey the actual number of immune patients. Considering only total anti-HAV detection to evaluate the immunity, no significant changes were found during the study period.

In 1984, 84.9% of the Portuguese population was immune to HAV and the prevalence of anti-HAV antibodies in patients less than 20 years old was 93.4% [12]. In 2002, the immunity had decreased to 58%, being 62.4% in patients less than 20 years old [22]. In this study, in the Aveiro District, the immunity rate was 22.1% for patients aged 6 to 15 years and 37.2% for patients aged 16 to 25 years. There was a great decrease in prevalence of anti-HAV antibodies throughout the last years for these age groups. According to World Health Organization (WHO), the estimated immunity rate in Western Europe countries was 18% for 10–14 years age group and 28% for 15–19 years age group, values that are even lower than those observed in Aveiro District [10]. The decrease in the immunity rate for the young patients can be explained by the improvement of sanitary conditions.

Although the rate of immunity increased with age, for the children groups it was quite low, under 25%, which could lead to an increased risk of unpredictable outbreaks in the future [8]. It is important to note that for children aged less than five years the seroprevalence was higher than for the older children. This fact can be explained by a higher number of vaccinated children relatively to the other groups, despite the HAV vaccine not being included in the National Plan of Vaccination [20]. For elderly people (>66) the immunity was lower than that observed for people aged 55 to 66 years old, what can be explained by the gradual weakening of the immune system with age. A mortality rate of symptomatic disease of 1.5/1000 between the children under five years of age and of 27/1000 in people aged over 50 years was observed [23]. Thus, these results bring a new concern, since the prevalence curve tends to deviate to the more advanced age groups [23].

The increase of immunity with age, not followed by an increase in the fraction of recently infected patients, suggests that patients contact with the viruses, acquiring immunity without symptoms. However, although the incidence of recent infection was low (only 22 cases), it is important to note that this study was made in ambulatory regime and usually people with symptoms of HAV infection are addressed directly to the hospital.

## 4. Materials and Methods

### 4.1. Samples

In this retrospective study all serum samples analyzed for total anti-HAV and IgM anti-HAV from patients of Aveiro District, in ambulatory regime, collected at the Clinical Analysis Laboratory Avelab (Aveiro, Portugal) during the period 2002–2012, were considered.

The age and sex of each patient were registered. The vaccination history of the patients was not available and could not be considered. The study was approved by the Ethical Committee of the Clinical Analysis Laboratory Avelab, specifically by the Pharmacist Doctor António Ferreira Neves and Doctors Alberto Ferreira Neves, António Rodrigues and Maria Teresa Raposo.

### 4.2. Sampling

Samples were collected using the Avelab Laboratory protocol. The venous blood of the patients was collected and reserved into a tube with separator spheres allowing clot formation. Each tube was centrifuged at 1381 × g for 10 min and placed in an automated system for the analysis. The analyses were done until one hour after collection. When this procedure was not possible, the samples were stored at 2–8 °C and processed until 24 h after collection. All the samples were dismissed seven days after sampling.

### 4.3. Antibodies Detection

The samples were analyzed in an automated Siemens ADVIA Centaur^®^ XP immunoassay analyzer. It was used the acridinium ester (AE) as the chemiluminescent label. 

Samples were diluted in buffer and purified HAV antigen was added forming in the presence of specific antibodies (IgG and/or IgM) immune complexes. AE labeled mouse monoclonal anti-HAV antibodies, biotinylated mouse monoclonal anti-HAV Fab fragment and streptavidin coated paramagnetic capture particles were incubated with the immune complexes. The biotinylated conjugate and acridinium labeled conjugate bind to antigen sites not occupied by sample HAV antibodies. A magnet is used to separate the microparticles and the unbound material is washed. The bound acridinium ester conjugate is then measured by a chemiluminescent reaction. The amount of light produced is inversely proportional to the antibodies concentration [24].

Total anti-HAV and/or IgM anti-HAV were analyzed in all samples. The presence of IgM anti-HAV indicates recently infected patients and the presence of total anti-HAV (IgG anti-HAV and IgM anti-HAV) indicates that patients had previous or ongoing infection.

The samples were classified as non-infected, recently infected, immune and non-immune, according to the detected antibodies. The patients were classified as immune when the result of total anti-HAV was positive and infected when the result of IgM anti-HAV was positive. Patients with negative anti-HAV antibodies (total anti-HAV negative and IgM anti-HAV negative) were considered non-immune and patients with negative IgM anti-HAV were considered non-infected.

### 4.4. Statistical Analysis

The data were treated using the Statistical Package for the Social Sciences (SPSS) 20.0 for Windows. To simplify the statistical analysis the patients were grouped by age ranges: 0–5, 6–15, 16–25, 26–35, 36–45, 46–55, 56–65 and >66. The absolute (n) and relative (%) frequencies were presented for qualitative variables. The normality of data was checked before analysis. As all the variables failed this statistical method assumption, the non-parametric Chi-square (X^2^) test was used to check if the distribution of variables was similar in the different groups for immunity. The significant level established was 0.05.

## 5. Conclusions

The results of this study suggest a positive impact of vaccination of children under five years old in the immunity pattern but the rate of immunity in the children groups are still quite low, indicating that the impact of future infections and outbreaks will be substantially more problematic in children than in adults. The decrease in the immunity in older patients, associated with the increase in the mortality rate in people over 50 years old, suggests that the impact of future infections will be also a concern for the elderly group. In the future, cost effectiveness studies of hepatitis A vaccine will be important to the decision of introducing universal vaccination of the Portuguese population, taking into account that some reports have shown its value in other developed countries.
